# Evaluating the Root Canal Morphology of Permanent Maxillary First Molars in Iranian Population 

**DOI:** 10.22037/iej.v12i4.17207

**Published:** 2018

**Authors:** Mohsen Rezaeian, Maryam Rouhani Tonekaboni, Foad Iranmanesh

**Affiliations:** a *Department of Epidemiology and Biostatistics, Occupational Environmental Research Center, Medical School, Rafsanjan University of Medical Sciences, Rafsanjan, Iran; *; b *Dentist, Rafsanjan University of Medical Science, Rafsanjan, Kerman, Iran; *; c *Department of Endodontics, Dental School, Rafsanjan University of Medical Science, Rafsanjan, Kerman, Iran*

**Keywords:** Maxillary First Molar, Root Canal Anatomy, Root Morphology

## Abstract

**Introduction:**

A successful endodontic treatment depends on a comprehensive knowledge of the morphology of canal and its variations, an appropriate access cavity, proper cleaning and shaping and adequate root canal filling. The present study was carried out to evaluate the root canal morphology of permanent maxillary first molars in an Iranian population.

**Methods and Materials:**

In this *in vitro* study, 80 extracted permanent maxillary first molars from a population in Rafsanjan, Iran were collected. Root canal morphology was evaluated by clearing technique under stereomicroscope under 40× magnification. A combination of Vertucci’s and Sert and Bayirli’s classifications were used to determine the root canal types. Data were analyzed by SPSS 18 software using descriptive statistics.

**Results:**

All palatal roots and almost all distobuccal roots had type I configuration. Ten different types of root canal system were found in mesiobuccal roots, among which type I was the most common (38.75%), followed by type II, IV, V, VI, IX, XV, XVI=XIX and VII, respectively**. **

**Conclusion:**

The mesiobuccal roots of permanent maxillary first molar had the most complex root configuration.

## Introduction

A successful endodontic treatment depends on a comprehensive knowledge of the morphology of canal and its variations, an appropriate access cavity, proper cleaning and shaping and adequate root canal filling. Sometimes a root canal may go undetected, which results in treatment failure [1]. Lack of knowledge about root canal morphology is the second cause of treatment failures [2, 3].

Several studies have analyzed the root canal morphology by different methods [4-12]. So, various root canal classifications have been developed based on these studies. The first classification was developed by Wein* et al.,* [4] in which the root canal systems were classified into four major types. Vertucci [5] classified the root canal systems into eight types using the clearing technique. Another study conducted on Burmese teeth added seven types to the previous classifications [6]. Sert and Bayirli [7] added fifteen new types of root canal systems to the Vertucci’s classification and came up with the total of 23 types of root canal systems.

Many studies have used different techniques to determine the root canal morphology, including radiography [8, 9, 13, 14], clearing technique [7-9, 15-20], cone-beam computed tomography (CBCT) [9, 10, 12, 13, 21-27] and micro computed tomography (micro-CT) [11, 28]. Although the clearing technique is a difficult and time-consuming process, it has some advantages such as preserving the original morphology of root canals, three-dimensional display of root canal, and show off lateral and accessory canals [4].

Several factors are involved in the variation of root canal systems. As an illustration, the ethnic effects on the variation of root canal system has been documented [29]. Furthermore, the maxillary first molar is one of the most complex teeth in root and canal morphology [1]. Up to now, numerous studies have examined the root canal morphology of maxillary first molars [5, 7-13, 15-25, 30, 31]. Besides, studies have assessed the impact of different races on the root canal morphology of permanent maxillary first molars [7, 8, 10-13, 15-25, 30].

The present study was aimed to evaluate the root canal system morphology of permanent maxillary first molars in a Rafsanjani population, by clearing technique according to the combination of Vertucci’s and Sert and Bayirli’s classifications of root canal systems.

## Materials and Methods

Eighty extracted permanent maxillary first molars were obtained from four clinics in Rafsanjan, a city in southeast of Iran. These teeth were extracted due to non-restorable caries or periodontal diseases. The roots were observed under dental loupes (Orascoptic, Middleton, WI, USA) to exclude those with cracks. The collected teeth were stored in distilled water (Farazmehr, Isfahan, Iran) until the start of laboratory procedures. Then, the teeth were kept in 10% formalin (Dr. Mojallali Industrial Chemical Complex Co., Tehran, Iran). 

To evaluate the root canal morphology, the clearing technique (Vertucci’s technique with few modifications) was used [5]. The crowns of the teeth were dissected from cemento-enamel junction by a disc (D & Z, Berlin, Germany). To prevent damage to root canal morphology and probable closure of the canals, no instrument was inserted into the canals in any stage of the procedure. 

**Figure1:**
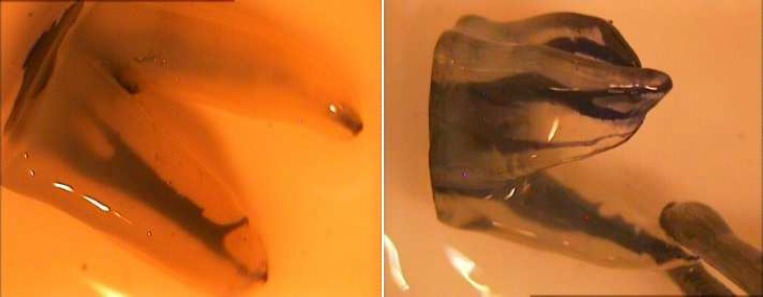
Samples were evaluated by stereomicroscope under 40× magnification. The mesiobuccal roots showed 10 different root canal configuration including type VI and type XVI

The samples were kept in 5% hydrochloric acid (Educational industries, Kerman, Iran) for 30 h and washed under tap water. The samples were then kept in 5% potassium hydroxide solution (Educational industries, Kerman, Iran) for 24 h and washed by tap water for 2. Then, hematoxylin dye (Farzaneh Arman Co., Tehran, Iran) was injected into the coronal pulp cavity by a micropipette (Isolab, Munich , Germany). The surgical suction (Houdian, Tehran, Iran) was used at the end of the roots in order to produce negative pressure. The teeth were kept dried in 70, 90 and 100% ethanol (ZakaryaJahrom, Jahrom, Iran) for 5 h, respectively. Finally, the samples were kept in transparent liquid polyurethane resin (Goharfam, Tehran, Iran) for 24 h to clear. The samples were evaluated by stereomicroscope (Olympus, Tokyo, Japan) under 40× magnification. A combination of Vertucci [5] and Sert and Bayirli [7] classification systems were used to determine the root canal morphology (Figure 1). Data were analyzed by SPSS software (SPSS version 18.0, SPSS Inc., Chicago, IL, USA).

## Results

In the present study, 80 permanent maxillary first molars were evaluated; 43 teeth (53.75%) from the male patients and 37 samples (46.25%) from the female patients. The patients’ age ranged from 21 to 68 with the mean age of 48 years.

Based on “Vertucci” and “Sert and Bayirli” classification in all roots, 10 different root canal types were detected. Type I was the most frequent type (79.16%) followed by type II, IV, V, VI, IX, XV, XVI=XIX and VII, respectively. 

All of the palatal roots showed type I root canal configuration. In the distobuccal (DB) roots, the most frequent type was type I (98.75%) and only one sample (1.25%) was type V. Moreover, 10 different types of root canals were seen in mesiobuccal (MB) roots. The most frequent type was type I (38.8%) followed by types II, IV, V, VI, IX, XV, XVI=XIX and VII, respectively (Table 1 and Figure 1).

## Discussion

The technique used in this study was the clearing technique. Although more advanced techniques, especially CBCT, have been widely used recently, the clearing technique is the most common method that is still being used for studying the root canal morphology [32]. Omer *et al.* [8] reported higher accuracy of clearing technique than radiography to determine root canal morphology. On the other hand, similar accuracy of clearing technique in comparison to CBCT and Peripheral Quantitative Computed Tomography (PQCT) have been reported [9].

The clearing technique used in the present study was performed with a minor modification compared to Vertucci’s technique. To minimize the chance of orifice/canal missing, the crowns were cut instead of preparing access cavity. This technique would leave the morphology intact and increase the chance of locating extra canals/orifices. Also, hydrochloric acid was used for 30 h instead of 24 h which created a proper decalcification without any damage (according to our pilot study). Since some root canals were narrow, a surgical suction was used to produce a negative pressure for a better penetration of dye into the canals.

Numerous studies [8, 12, 13, 16, 17, 19-22, 24, 30] used Vertucci's classification but Sert and Bayirli's classification has fifteen additional canal types [7], and only one study [18] used this classification. Two studies [15, 23] used Vertucci's classification but found additional canal types which can be found in the Sert and Bayirli's study [7]. Therefore, the most comprehensive classification was used in present study: a combination of Vertucci and Sert and Bayirli.

In all roots, the most common canal configuration was type I. This finding is in coordinate with the results of many studies with different races [7, 15, 18, 21-24]. Therefore, it seems that this finding is reliable for the permanent maxillary first molar regardless of race or methodology.

In MB roots, the most common canal configuration was type I. A review study shows that the Vertucci type I was the most common type (35.70 %) of MB roots of maxillary first molar in Iranian population [33]. Furthermore, Rouhani *et al.* [24], Neelakantan *et al.* [23] and Rwenyonyi *et al.* [18] studies show the same results. But Faramarzi *et al.* [21] and Khademi *et al.* [12] found that type II was the most common type of root canal in MB roots, followed by type I. In the studies by Zand *et al.* [13], Naseri *et al.* [22], Sert *et al.* [7], and Alavi *et al.* [15], the most common type was III, VI, II and IV, respectively. However; in Zand *et al.* [13] study, the difference between type III and type I was negligible. Considering the most common canal type in MB roots, the mentioned studies [7, 12, 13, 15, 18, 21-24] have diverse results. These studies were carried out on different populations, including Iranian [12, 13, 21, 22, 24], Indian [23], Ugandan [18], Turkish [7] and Thai [15], and also were done with two different methods, including CBCT [12, 13, 21-24] and clearing technique [7, 15, 18]. Therefore, it is difficult to relate these various results to race or method. 

Furthermore, in MB roots, there were ten different root canal types, four of which belonged to Sert and Bayirli’s classification. Unfortunately, most studies [12, 13, 15, 21-24] are based on the Vertucci’s classification, so a comprehensive comparison is not possible. Rouhani *et al.* [24] had seven different Vertucci’s types with an additional type (1-3-2-1) which did not belong to any classification. Alavi *et al. *[15] and Neelakantan *et al.* [23] were done based on Vertucci’s classification. Rwenyonyi *et al.* [18] used Sert and Bayirli’s classification, but they only found one type apart from the types seen from the Vertucci’s type. In the other studies [7, 12, 13, 21, 22, 34], the diversity of canal type was limited to the Vertucci’s classification. Variety of root canals and their prevalence, were not similar in different studies. These differences may be attributed to different methodologies, sample size or race (Table 2). 

**Table 1:** Percentage and morphology variations of mesiobuccal root canal system

**Canal type**	**I**	**II**	**IV**	**V**	**VI**	**VII**	**IX**	**XV**	**XVI**	**XIX**	**Total**
**N (%)**	31 (38.75)	13 (16.25)	11(13.75)	7(8.75)	6(7.5)	1(1.25)	4(5)	3(3.75)	2(2.5)	2(2.5)	80(100)

**Table 2:** Variety of mesiobuccal roots canal types in present and other similar studies

**Study**	**Faramarzi ** ***et al. *** **[21]**	**Zand ** ***et al. *** **[13]**	**Rwenyonyi ** ***et al.*** **[18] **	**Naseri ** ***et al. *** **[22]**		**Sert ** ***et al.*** ** [7] **		**Alavi ** ***et al. *** **[15] **		**Neelakantan ** ***et al.*** ** [23] **	**Khademi ** ***et al.*** ** [12] **		**Present study**	
**Population**	Iranian	Iranian	Ugandan		Iranian		Turkish		Thai	Burmese		Iranian		Iranian	
**Method**	CBCT	CBCT	clearing		CBCT		clearing		clearing	CBCT		CBCT		clearing	
**I**	30.77	43.6	75.1		13.4		6.5		32.7	51.8		29.8		38.75	
**II**	49.35	10.3	4.1		32.9		39.5		17.34	5.5		53.1		16.25	
**III**	0	44.2	0.9		1.3		14.5		1.9	0		0		0	
**IV**	19.87	0.6	11.3		11.4		28		44.2	38.6		0.77		13.75	
**V**	0	1.3	5.8		5.4		2		1.9	0		0.25		8.75	
**VI**	0	0	1.4		35.68		3		0	0		0		7.5	
**VII**	0	0	0.9		0		5.5		0	0		0		1.25	
**VIII**	0	0	0		0		0		0	0		0		0	
**IX**	0	0	0.5		0		0		0	0		0		5	
**XV**	0	0	0		0		0		0	0		0		3.75	
**XVI**	0	0	0		0		0		0	1		0		2.5	
**XVII**	0	0	0		0		0		1.9	0		0		0	
**XIX**	0	0	0		0		0		0	0		0		2.5	

In DB roots, type I was the most common root canal type in the present study. All other studies which evaluated DB roots canal types [7, 15, 18, 21-24] have shown the same results; however, the prevalence was not the same. The prevalence range was from 89.9% [22] to 100% [21]. Therefore, it seems that this finding is reliable for the DB roots of permanent maxillary first molars regardless of race or methodology.[15], [18] and [21]. Moreover, other studies [7, 9, 22, 24] show the same results, but with different prevalence (from 88.1 to 96.6%). However, an unusual canal morphology in the palatal root may occur [35].

Finally, in palatal roots, all root canal configurations were type I. This finding is in line with Alavi *et al.* [15], Rwenyonyi *et al.* [18] and Faramarzi *et al.* [21]. Moreover, other studies [7, 22-24] show the same results, but with different prevalence (from 88.1 to 96.6%). It seems that the results of the present study is reliable regardless of race or methodology. However, an unusual canal morphology in the palatal root may occur [35].

## Conclusion

Within the limitations of this study, it can be concluded that in the permanent maxillary first molars, mesiobuccal roots have a more complex root canal configuration than palatal and distobuccal roots.
